# Of Toasters and Molecular Ticker Tapes

**DOI:** 10.1371/journal.pcbi.1002291

**Published:** 2011-12-29

**Authors:** Konrad P. Kording

**Affiliations:** Northwestern University, Departments of Physical Medicine and Rehabilitation, Physiology, and Applied Mathematics, Chicago, Illinois, United States of America; University College London, United Kingdom

## Abstract

Experiments in systems neuroscience can be seen as consisting of three steps: (1) selecting the signals we are interested in, (2) probing the system with carefully chosen stimuli, and (3) getting data out of the brain. Here I discuss how emerging techniques in molecular biology are starting to improve these three steps. To estimate its future impact on experimental neuroscience, I will stress the analogy of ongoing progress with that of microprocessor production techniques. These techniques have allowed computers to simplify countless problems; because they are easier to use than mechanical timers, they are even built into toasters. Molecular biology may advance even faster than computer speeds and has made immense progress in understanding and designing molecules. These advancements may in turn produce impressive improvements to each of the three steps, ultimately shifting the bottleneck from obtaining data to interpreting it.

**This is an “Editors' Outlook” article for *PLoS Computational Biology***

Moore's law has characterized progress in microprocessor techniques (see Figure 1, dashed). In very good approximation, computers have doubled in speed every 2 years. At the same time, computing has progressively gotten cheaper, and we can now successfully solve many computing problems that once seemed hard, such as speech recognition. We are also solving entirely new computational problems, such as searching billions of web pages for information on neuroscience or molecular biology. Such exponential growth makes solutions to seemingly insurmountable problems seem trivial given a bit of time.

**Figure 1:**
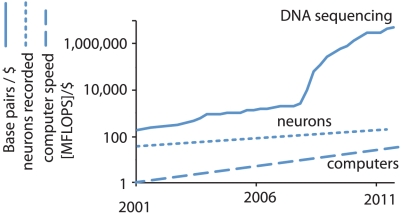
Comparison of the scaling laws between neuroscience (exponential fit from [1]), computer science (exponential fit of years 2000–2007 from [29]), and DNA sequencing, see [30]. Remarkably, the steepness of the curves is worth comparing while the offset on the *y*-axis is arbitrary.

Meanwhile, simple computers have gotten cheaper over time. This decrease in costs was so dramatic that many of today's toasters contain microprocessors for time-keeping, switching on or off heat, light feedback of heating state, and the handling of key presses. This makes building toasters simpler and ultimately cheaper. When computers were invented, toasters were certainly not an expected application of computing.

Importantly, the speed of computers has increased dramatically faster than the number of neurons that can be simultaneously recorded [1] (Figure 1, dotted). Still, the increase of the number of simultaneously recorded neurons has allowed the development of advanced algorithms that take advantage of this growing number. Indeed, the field that analyzes multivariate neural data is large now and can analyze complicated interactions between large numbers of neurons [2]–[4], electroencephalography (EEG) or magnetoencephalography (MEG) recordings, optical recordings, or functional magnetic resonance imaging (fMRI) voxels [5], [6]. However, as the amount of data increases, so does the complexity of the questions. For example, many current studies of neural data analysis ask how neurons interact, but as the number of neurons grows, the number of potential connections grows quadratically as each neuron may interact with each other neuron. This, in turn, leads to models with many free parameters, which requires new statistical methods of fitting these parameters.

Molecular biology has seen accelerating progress over the last decades. One readily quantifiable cost in molecular biology is that of sequencing DNA. The development of a host of different methods has allowed the cost of sequencing each base pair to dramatically decrease over time (Figure 1, solid). The rate of improvement is much faster than that of neuron recording techniques or even Moore's law. This development allowed sequencing the entire human genome at a price of billions of dollars in the year 2003, and sequencing the same genome at higher quality now costs less than $2,000. The current push is to sequence an entire genome for less than $1,000 [7]. This development allows solving many problems of obvious importance, such as the search for gene-related markers of disease [8].

From a computational perspective, a central objective of neuroscience is to understand how neurons convert their inputs into outputs and collectively produce action based on stimuli and internal processes, such as memory and attention. This leads to what I would call the three central steps of experimental approaches in systems neuroscience. (1) Select the signals that are important for a given neuroscience question. As long as we cannot approach understanding the entire brain at the same time, it is highly useful to select what to stimulate and what to measure. (2) Get stimuli into the brain. To understand what neurons do, inputs need to be defined or known. (3) Get data out of the brain. Only large amounts of data allow meaningful statistical inferences. Virtually all experimental approaches to systems neuroscience can be phrased in these terms.

It is interesting to ponder a few well-known examples. In a typical single-cell visual cortex experiment [9] that studies how the visual cortex encodes visual stimuli, we would put an electrode into primary visual cortex (1), show various visual stimuli on a monitor in front of the animal (2), and record neural activity from the electrode (3). In a typical fMRI experiment about visual cortex [10], we would choose a contrast that tells us about changes in blood flow in the brain (which is a proxy for average firing rate) (1), stimulate subjects by using a set of fixed visual stimuli (2), and read out data using resonance signals (3). In a typical slice work experiment [11], we might identify a specific cell type under the microscope (1), activate other cells using glutamate uncaging (2), and then record signals from the intracellular electrode to find out how the other cells affect the recorded cell (3). In all these cases, selection, stimulation, and reading the data (“data out”) are crucial aspects of the work.

Each of these steps has its own criteria for being maximally useful. For the selection step (1), we would like to select all the relevant signals and nothing else. For the stimulation step (2), we want to be able to get in large amounts of well defined data with high information bandwidth and low noise [12]. Lastly, for the “data out” step (3), we want to get a lot of data out with low noise and high bandwidth. Lastly, given limited budgets, we want the techniques to be cheap. Certainly, as we scale up the analysis towards understanding larger systems, the price per unit of information must be limited.

Each of the currently used approaches has limitations along the three steps. For example, fMRI has a huge number of channels, and can select essentially any brain area. Yet, it seems unlikely that it could be used to record only from certain cell types, such as interneurons [13], noise levels are high, and spatiotemporal resolution is low. Single-cell slice physiology is good at selecting neurons based on observable features like brain region and cell morphology, and it is low noise, but data out bandwidth is quite low and there may be biases in the selection of the recorded cells. Similarly, other current techniques are all rather limited on at least one of the three axes.

Molecular biology is starting to offer powerful solutions to overcome limitations of the three steps [14], and I want to start by reviewing past progress.

(1) The selection step. There are many different levels of selection. We might want to select individuals that have certain diseases for which there are genetic markers. In this case, we can select these individuals through genetic tests [15]. We might want to select neurons but not other cells (Figure 2A, upper), and this is possible through a set of well-established genetic methods that enable or disable gene expression using tissue-specific promoters [16], [17]. We might want to select only a subset of all the cells (Figure 2A, middle) [18]. Lastly, we might only want to only select certain neurons that have defined physiological properties (Figure 2A, lower). Interestingly, it is even possible to select each cell individually and assign it a random color that is visible under the microscope [19]. Importantly, genetically selecting neurons enables certain ways of stimulating just those neurons and reading out only from those neurons (see below).

**Figure 2:**
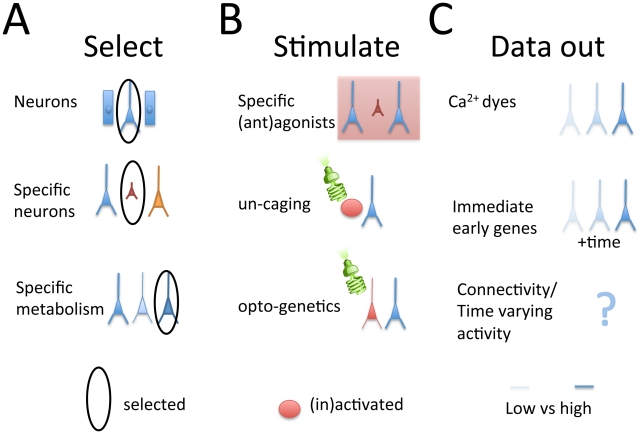
The three central steps that define experiments in systems neuroscience. The question mark denotes areas where the author expects exciting developments. (A) Methods for selecting where neural signals come from. (B) Methods for stimulating neurons. (C) Methods for reading out the data. See text for detail.

(2) The stimulation step. Neurons are traditionally probed with external stimuli (e.g., images on computer monitors), or through electrical or magnetic stimulation methods. Molecular approaches, though relatively new and less frequently employed, allow several clear advantages. First, they can be utilized far more selectively. For example, specific ligands can selectively activate or inactivate certain cellular mechanisms, including those that only exist in certain types of neurons (Figure 2B, upper). They allow activating neurons upon light stimulation, e.g., through glutamate uncaging [20] (Figure 2B, middle). The selection techniques (see above) allow making it so that stimulation will only affect neurons or even only selective subsets of neurons. For example, standard genetic techniques allow inactivating certain neurons [21] or activating them later in life upon injection of a drug [22]. More recently, optogenetic techniques (Figure 2B, lower) have allowed selectively activating or inactivating specific neuron types at specific points in time [23], [24].

(3) The “data out” step. Getting data out of neurons is traditionally done using either existing signals (as in intrinsic imaging), secondary signals like blood flow (as in fMRI), dyes (as in calcium imaging), or electrical or magnetic recording (as in microelectrodes and MEG signals). Using molecular techniques, it is possible to have neurons express the dye used to monitor them. Molecular approaches promise some advantages due to the possibility of “clean” selection (see above (1)). For example, it is possible to express a dye only in those neurons of interest (Figure 2C, upper). Improving upon the basic functionality of dyes, it is possible to use the concentration of activity-dependent molecules to estimate activities. For example, it is known that immediate early genes' RNAs change their distribution within each neuron over time after activation [25]. This allows distinguishing the neural activity immediately preceding the end of the experiment from neural activity that happened earlier in the experiment. It thus provides some indication of neural activation patterns over time (Figure 2C, middle) and allows the visualization, within a single brain, of different neuronal populations engaged by two distinct experiences.

All these developments in molecular biology already make it a major driving force in neuroscience. However, in the same way that exponential growth in computer science has brought us better toasters, I expect that molecular biology will provide refinements of the three steps that are currently hard to imagine. Some past predictions of computer abilities (e.g., robot control) have been rather unimpressive, whereas others have been rather precise (e.g., Moore's law). While I am a computational neuroscientist with limited background in molecular biology, I still want to go out on a limb and make some predictions of developments we may see.

The readout of data is currently done using physics, thin wires, and optics, and it may be expected that molecular approaches, aided by the decay in cost of DNA sequencing, may offer new approaches. I can see two major classes of experimental questions. Connectivity: I want to know how neurons and brain areas are wired up [26]. Activity: I want to know how each neuron's firing relates to outside variables such as movement or perceptual stimuli and to other neurons. If these two problems could be reduced to DNA sequencing, then progress in molecular approaches may well lead to a new class of approaches to the data-out step. In particular, these approaches seem desirable because of synergies with ongoing molecular improvements over the other two steps.

To solve the connectivity problem, Tony Zador [27] proposed a sequencing-based solution. Every neuron would have its own unique, random DNA sequence barcode tag transportable to all post-synaptic neurons using a transsynaptic virus. All the tags of presynaptic neurons would then be fused into a long string of DNA that would be read through DNA sequencing. If every neuron has a unique tag (i.e., the tag is random and long enough) and the trans-synaptic transport works without faults, then a unique identification of the brain's connectivity would be possible; otherwise, statistics will allow a probabilistic identification.

Lastly, it seems that the step of recording neural activity can also be reduced to DNA sequencing. When a cell divides, it naturally copies its entire DNA using DNA polymerase. The movement of the polymerase along the DNA template could be engineered to be essentially a molecular ticker tape, such that the environment at that point in time is recorded in the DNA sequence (for details on potential molecular implementations, see [28]). This could be achieved by engineering a polymerase that would make errors when neural activities are high, for example, such errors could be modulated by calcium concentration. While copying a template, DNA polymerase could thus write the temporal trace of activity as error patterns onto DNA molecules (see Figure 3). Of course, these would be difficult steps, and neither DNA polymerase that depends on neural activity nor steady template copying in quiescent neurons has been established. Still, the sketched approach could in principle allow high temporal resolution combined with very high spatial resolution.

**Figure 3:**
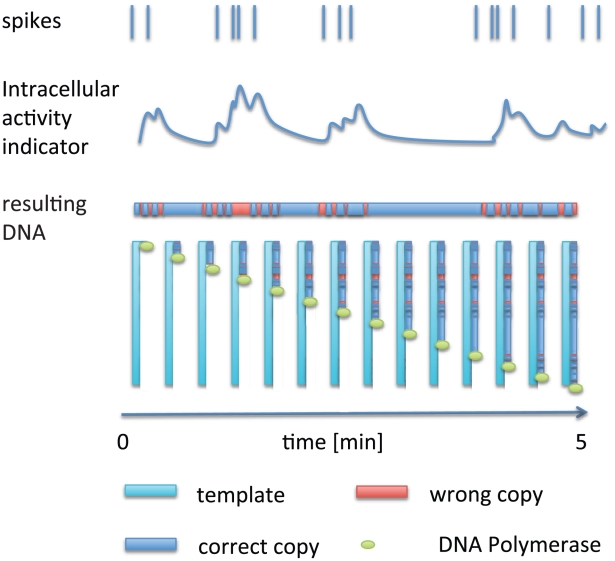
Molecular ticker tapes. Neural activity affects an intracellular concentration. DNA polymerase copies a template with a fidelity that is regulated by an indicator for neural activity. Sequencing thus yields the indicator concentration as a function of time, and therefore the activity.

Molecular biology is making rapid progress at becoming useful for systems neuroscience. So far, there have been outstanding approaches at improving the selection step—many types of neurons can be selected individually. The stimulation step has been affected by techniques that allow impressive precision. Data out is a promising field, and it seems that molecular biology will have its strongest impact if it combines strong solutions to all three steps. From a systems neuroscience perspective, molecular developments are going to produce large amounts of highly relevant information. In the same way that microprocessors made their way into our toasters and made them better and cheaper, we now can see how custom-designed molecular machines may make experiments in system neuroscience cheaper and more powerful. However, it seems important to realize that the development of all these tools has high promise—but ultimately, data does not suffice to understand how we perceive, think, and act. If molecular techniques allow massive amounts of data about the brain to be obtained, the central problem will be how to interpret and make sense of this data, a problem similar to other Omics approaches. Cheaper experiments will lead to massive amounts of data furthering an ongoing shift from obtaining data to interpreting it.

**Author's Biography**

**Konrad Kording** has a PhD in physics from the Federal Institute of Technology (ETH), Zurich, Switzerland, where he worked on cat electrophysiology and neural modeling. He received postdoctoral training in London where he worked on motor control and Bayesian statistics. Another postdoc, partially funded by a German Heisenberg stipend, followed at MIT where he worked on cognitive science and natural language processing and deepened his knowledge of Bayesian methods. Since 2006 he has worked for the Rehabilitation Institute of Chicago and Northwestern University, where he received tenure and promotion to associate professor in 2011. His group is broadly interested in uncertainty, using Bayesian approaches to model human behavior and for neural data analysis. Dr. Kording is a Deputy Editor with *PLoS Computational Biology*.
